# Comparison of propofol-esketamine versus propofol for anesthesia in gastroscopy: a double-blind, randomized controlled clinical trial

**DOI:** 10.3389/fmed.2023.1184709

**Published:** 2023-08-08

**Authors:** Xiaoli Liu, Qingyu Xiao, Shaohui Zhuang

**Affiliations:** Department of Anesthesiology, The First Affiliated Hospital of Shantou University Medical College, Shantou, Guangdong, China

**Keywords:** esketamine, propofol, gastroscopy, anesthesia, hemodynamic stability

## Abstract

**Objective:**

To compare the effects of propofol-esketamine and propofol in gastroscopy in adults.

**Methods:**

This randomized controlled clinical trial was performed from January 2021 to March 2021. Eighty patients were enrolled and allocated into normal saline group (group N) and esketamine group (group E). The primary outcome was total amount of propofol. Secondary outcomes included incidences of injection pain, involuntary movement, hemodynamic and respiratory adverse events during examination, total examination time, recovery time and postoperative adverse effects.

**Results:**

Total amount of propofol was significantly smaller in group E (101.64 ± 32.64 mg) than in group N (129.55 ± 36.34 mg, *p* = 0.001). Incidences of injection pain, involuntary movement and hypotension was significantly lower in group E than in group N. Incidences of hypertension and tachycardia was higher in group E than in group N. There was no significant difference in incidences of laryngospasm or hypoxemia, total examination time, recovery time, incidences of postoperative adverse effects between two groups.

**Conclusion:**

Combination of propofol with 0.2 mg/kg esketamine reduced total amount of propofol, provided a more stable hemodynamic status and did not affect recovery time in gastroscopy.

**Clinical trial registration:**

http://www.chictr.org, identifier ChiCTR2100042406.

## 1. Introduction

Gastroscopy, as flexible endoscopy, has become a common diagnostic and therapeutic method for gastrointestinal disease due to its characteristics of no pain, small trauma, quick operation, and accurate diagnosis (1). Propofol has the advantages of short onset time, fast recovery, complete metabolism, and short half-life, which is generally applicable to be used as a sedative combined with other analgesics in gastroscopy (2–4). However, extensive research has shown that propofol sedation can influence the stability of respiration and circulation, increase adverse reactions, such as hypoxemia and hypotension (5).

In the past few decades, ketamine has been generally used for its potent sedative and analgesic effects but has gradually been limited because of psychiatric side effects (6, 7). Esketamine, an S-isomer of ketamine and an antagonist of N-methyl-D-aspartate receptors, is twice as potent as racemic ketamine (8, 9) with both sedative and analgesic effects, but few adverse reactions (10–12). The aim of this study is to compare the effect of propofol-esketamine versus propofol for anesthesia in gastroscopy.

## 2. Materials and methods

### 2.1. Ethics approval

This trial was approved by the Medical Ethics Committee of the First Affiliated Hospital of Shantou University Medical College. All recruited patients signed the informed consent. The study was registered at Chinese Clinical Trial Registry on January 11, 2021 (http://www.chictr.org, registration number: ChiCTR2100042406).

### 2.2. Sample size estimation

Sample size was calculated by a preliminary study in which 20 patients were included and allocated into normal saline group (group N) and esketamine group (group E). Total amount of propofol was 112.350 ± 6.324 mg in group N and 106.500 ± 9.437 mg in group E. Thirty-one patients in each group were required to obtain a two-sided significance level (α) of 0.05 and power (β) of 80%. Assuming a loss to follow-up rate of 15%, 40 patients in each group (80 in total) were enrolled.

### 2.3. Participants

Patients who underwent gastroscopy in the First Affiliated Hospital of Shantou University Medical College from January 2021 to March 2021 were selected. Patients aged 18–64 years with ASA grading of I to III and BMI no more than 30 kg/m^2^ were included in the study. Patients allergic to ketamine or propofol; with chronic heart failure or atrial fibrillation, severe liver or kidney dysfunction, history of mental illness or central nervous system disorders, treatment with hypnotics or analgesics in the past 3 months, uncontrolled hypertension, increased intracranial pressure, hyperthyroidism or glaucoma were excluded.

### 2.4. Randomization and blinding

Randomization was performed through an interactive web response system (Brightech Clinical Information Management System). In order to increase the comparability between groups and prevent severe imbalances caused by possible confounders (13, 14), patients were randomly assigned (1,1) to normal saline group (group N) and esketamine group (group E) using a computer-generated random numerical series and block randomization (block size of four). Then group information was kept in an opaque envelope.

Before gastroscopy examination, anesthesiologist A (Dr. Jiamei He) obtained the group information from the opaque envelope and prepared the medication. The solutions were configured with normal saline to 10 mL which appeared colorless and odorless. The 10 mL transparent syringe without any label was put inside a box together with propofol for the recruited patient. After medication preparation, Dr. He left the examination room without any communication with the investigator or the endoscopist. Anesthesiologist B (Dr. Qingyu Xiao) was responsible for intravenous medication administration. After infusion of 10 mL liquid (esketamine or normal saline), she administered initial dose and extra doses of propofol until the patient lost consciousness. Data collection was done by anesthesiologist C (Dr. Xiaoli Liu) who was also blinded to group assignment and recorded vital signs at specific timepoints regardless of the medication administration process. Amount of propofol was recorded at the end of the examination. The endoscopist or the patient were not aware of group assignment either.

### 2.5. Study design

Venous line was obtained on the right upper extremity before examination. Patients lied down in a left decubitus position. Oxygen with a flow rate of 2–3 L/min was supplied via a nasal cannula. Patients in group E were administered 0.2 mg/kg esketamine (Jiangsu Hengrui, SFDA approval No. H20193336) intravenously, while patients in group N were administered normal saline. One minute later, initial dose of 1 mg/kg propofol (Diprivan SFDA approval No. H20171275) was administered intravenously (15). Then the anesthesiologist called the patient’s name. If the patient was still awake or body movement was noted, extra doses of 0.5 mg/kg propofol was repeated until the patient lost consciousness without body movement (16). Eyelash reflex was finally tested for conformation of unconscious state, which allowed insertion of gastroscope (17, 18).

All examinations were performed by one experienced endoscopist (Dr. Yu Zhang). During examination, an extra dose of 0.5 mg/kg propofol was added if patients showed involuntary movement or swallowing reflex (17, 19).

Heart rate (HR), mean arterial pressure (MAP) and pulse oximetry (SpO_2_) were recorded before induction (T0), right after administration of normal saline or eskemine (T1), right after administration of initial dose of propofol (T2), when gastroscope entered the first narrowing of esophagus (T3), when gastroscope entered the duodenum (T4), when gastroscope exited the pharynx (T5) and when the patient opened the eyes for the first time during the recovery period (T6). Total examination time was defined as the time duration from T1 to T5. Recovery time was duration from T5 to T6.

Intraoperative adverse events included injection pain, involuntary movement, hypertension, hypotension, tachycardia, bradycardia, laryngospasm and hypoxemia. Hypertension or hypotension was defined as systolic blood pressure (SBP) increased or decreased by more than 25% of the first recorded SBP in the examination room. Tachycardia or bradycardia was defined as heart rate (HR) increased or decreased by more than 25% of the first recorded HR on the monitor screen (20) Urapidil (10–20 mg) or ephedrine (0.1–0.2 mg/kg) was administered IV to treat hypertension or hypotension, respectively. Esmolol (1 mg/kg) or atropine (0.005–0.01 mg/kg) was administered IV to treat tachycardia or bradycardia, respectively (16, 21). Hypoxemia was defined as 75% < SpO_2_ < 90% in 60 s or SpO_2_ < 75% at any moment (20, 22). In case of hypoxemia, rescue methods including increase of oxygen flow rate, mask ventilation or endotracheal intubation were taken immediately according to specific clinical situations.

During recovery period after the examination, the investigator called the patient’s name in a normal tone every 10 s. Observer Assessment of Alertness/Sedation Scale (OAA/S) score and the Modified Post-Anesthesia Discharge Scoring System–Modified (PADSS) score were evaluated every 5 min from the moment the patient opened the eyes. When the OAA/S score was no less than 4 and PADSS score was no less than 9, patients were asked about adverse effects including nausea, vomiting, dizziness, headache, drowsiness and excessive dreams. If no uncomfortness was complained, they were allowed to return to the ward accompanied by a nurse.

On the first day after the examination, the investigator followed up the patients through telephone to record any adverse effects after going back to the ward. Visual Analogue Scale (VAS) scores after examination and satisfaction scores were also recorded.

### 2.6. Outcome assessment

The primary outcome was total amount of propofol, meaning the sum amount of the initial dose during induction and the cumulated extra doses during gastroscopy. Secondary outcomes were incidences of intraoperative adverse events, including injection pain, involuntary movement, and hemodynamic adverse events like hypertension, hypotension, tachycardia, and bradycardia, respiratory adverse events like laryngospasm and hypoxemia; incidences of postoperative adverse effects including nausea, vomiting, dizziness, headache, drowsiness, and excessive dreams. Total examination time, recovery time, OAA/S, PADSS, VAS and satisfaction scores were also recorded.

### 2.7. Statistical analysis

SPSS 26.0 was used for data analysis. Kolmogorov–Smirnov test was applied to determine whether continuous variables were normally distributed. Continuous data were expressed as mean ± standard deviation (SD) with a normal distribution or as median (interquartile range) with a non-normal distribution. Total amount of propofol, total examination time and recovery time were analyzed applying a two-sample independent *t* test or Mann–Whitney U test. Incidences of intraoperative adverse events and postoperative adverse effects were compared using chi-square test or Fisher’s exact test. Therapeutic effect was accessed using odds ratio (OR) and 95% CI. A two-tailed value of *p* < 0.05 was considered statistically significant.

## 3. Results

**Figure 1:**
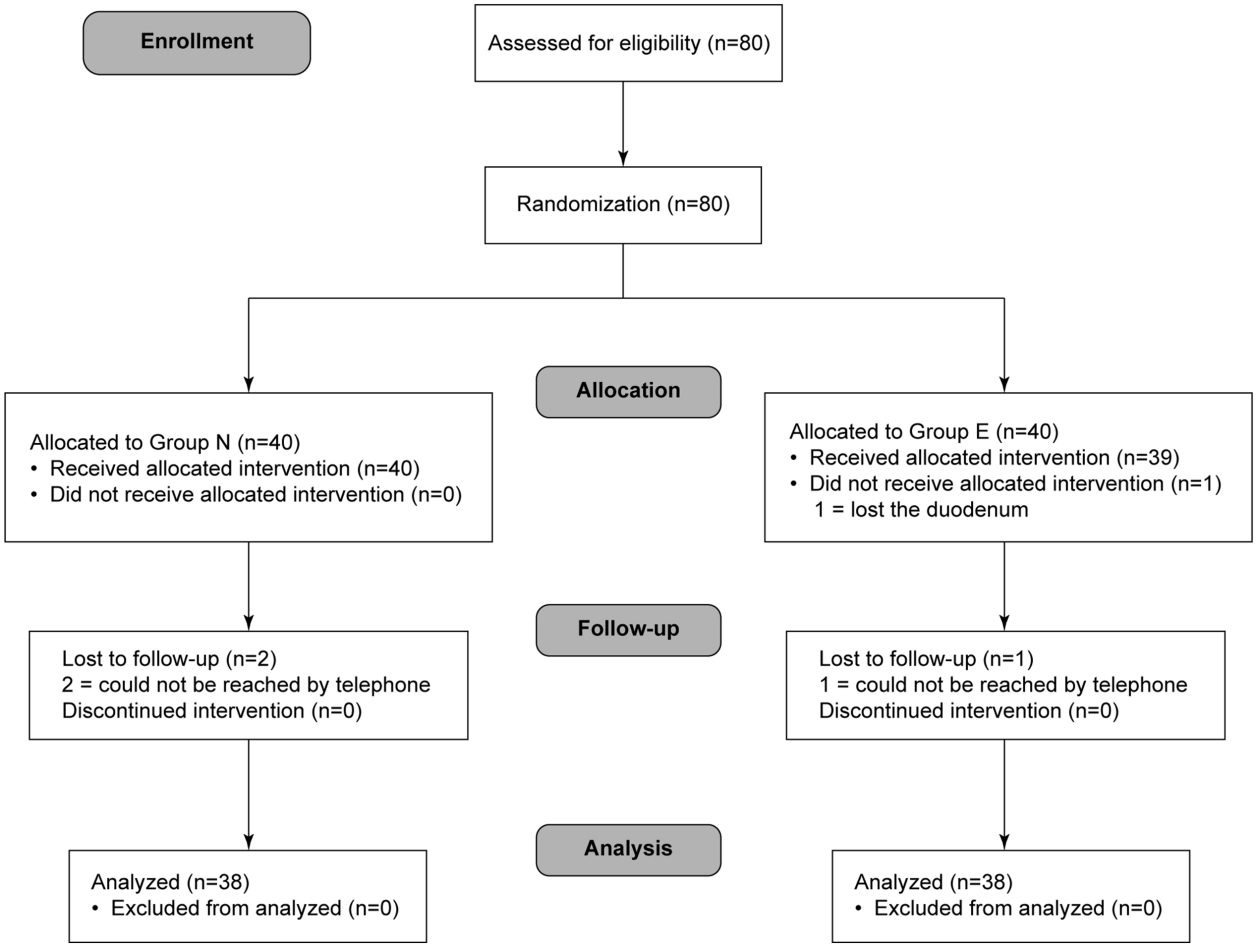
Flow chart of participants in the trial.

A total of 76 patients completed the study protocol (38 in group N and 38 in group E) (Figure 1). General conditions were not different between two groups (Table 1).

**Table 1:** Patient baseline characteristics.

	Group N (*n* = 38)	Group E (*n* = 38)	p^*^
Age (years)	49.03 ± 10.81	45.68 ± 13.83	0.360
Gender (male/female)	12/26	16/22	0.342
Height (cm)	161.29 ± 8.13	163.66 ± 6.99	0.178
Weight (kg)	58.71 ± 11.51	57.84 ± 9.41	0.720
BMI (kg/m^2^)	22.40 ± 3.07	21.57 ± 3.02	0.237
ASA-PS (I/II/III)	28/9/1	28/9/1	>0.999
Mallampati score (I/II/III/IV)	20/10/6/2	16/14/8/0	0.398
OAA/S score	5 (5, 5)	5 (5, 5)	>0.999
VAS score	0 (0, 1)	0 (0, 1)	0.728
Stomach diseases diagnosed by gastroscopy	25 (65.79)	27 (71.06)	0.622
Acute/Chronic gastritis	11 (44.00)	12 (44.44)	0.803
Gastric polyps	10 (40.00)	11 (40.74)	0.798
Gastric ulcer	3 (12.00)	2 (7.41)	0.644
Others	1 (4.00)	2 (7.41)	0.556

BMI, body mass index; ASA-PS, American society of anesthesiologists physical status; OAA/S, observer assessment of alertness/sedation scale; VAS, visual analogue scale. Data are presented as mean + SD or median (range) or *n* (%). ^*^Significance level (0.05).

There was no significant difference in initial amount of propofol between the two groups. Additional amount and total amount of propofol was also significantly smaller in group E than in group N (Table 2).

**Table 2:** Consumption of propofol.

	Group N (*n* = 38)	Group E (*n* = 38)	p^*^
Initial amount of propofol (mg)	58.71 ± 11.51	57.84 ± 9.41	0.720
Additional amount of propofol (mg)	70.84 ± 32.07	43.80 ± 30.12	**<0.001**
Total amount of propofol (mg)	129.55 ± 36.34	101.64 ± 32.64	**0.001**

Data are presented as mean ± standard deviation (SD) and analyzed using a two-sample independent *t* test or Mann–Whitney U test. ^*^Significance level (0.05). Bold numbers mean statistically significant *p* values.

**Figure 2:**
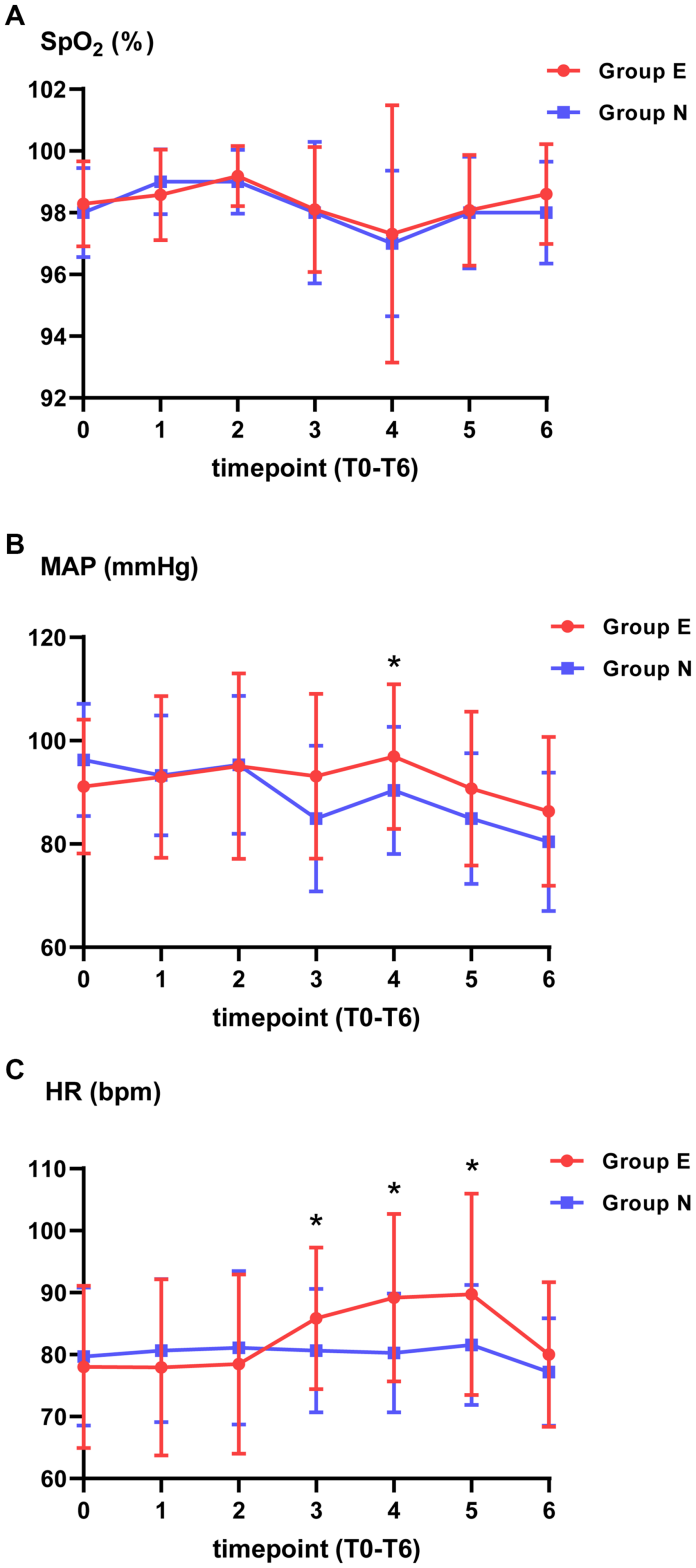
Vital signs at different timepoints. **(A)** Pulse oximetry (SpO_2_). **(B)** Mean arterial pressure (MAP). **(C)** Heart rate (HR). ^*^p value is statistically significant between two groups.

There was no significant difference in SpO_2_ between two groups at each timepoint. Compared with group N, MAP in group E was higher at T4. HR was higher in group E than in the group N at T3, T4 and T5 (Figure 2).

Incidences of injection pain, involuntary movement and hypotension was significantly lower in group E than in group N. Incidences of hypertension and tachycardia was higher in group E than in group N. No patients experienced laryngospasm. The incidence of hypoxemia did not differ between the two groups (Table 3).

**Table 3:** Incidence of intraoperative adverse events.

	Group N (*n* = 38)	Group E (*n* = 38)	Adjusted OR (95% Cl)	p^*^
Injection pain	13 (34.21)	5 (13.15)	0.291 (0.092, 0.925)	**0.031**
Involuntary movement	30 (78.94)	21 (55.26)	0.329 (0.120, 0.903)	**0.028**
Hypotension	14 (36.84)	2 (5.26)	0.095 (0.020, 0.457)	**0.001**
Hypertension	0	9 (23.68)	N/A	**0.005**
Tachycardia	3 (7.89)	19 (50)	11.667 (3.056, 44.539)	**<0.001**
Bradycardia	1 (2.63)	0	N/A	>0.999
Laryngospasm	0	0	N/A	>0.999
Hypoxemia	3 (7.89)	5 (13.15)	1.768 (0.391, 7.988)	0.709

N/A, not applicable. Data are presented as *n* (%) and analyzed using chi-square test or Fisher’s exact test. Therapeutic effect is accessed using odds ratio (OR) and 95% CI. ^*^Significance level (0.05). Bold numbers mean statistically significant *p* values.

There was no significant difference in total examination time or recovery time between two groups (Table 4).

**Table 4:** Duration of anesthesia.

	Group N (*n* = 38)	Group E (*n* = 38)	p^*^
Total examination time (s)	538.36 ± 169.59	471.60 ± 124.06	0.054
Recovery time (s)	180.11 ± 98.35	222.66 ± 130.79	0.113

Data are presented as mean ± standard deviation (SD) and analyzed using a two-sample independent *t* test or Mann–Whitney U test. ^*^Significance level (0.05).

Table 5 demonstrated the follow-up data. No difference was found in incidences of postoperative adverse effects. No patients were diagnosed with delirium postoperatively. OAA/S score and PADSS score between two groups at 5, 10 and 15 min after patients opened the eyes were not different (*p* > 0.05 for all). In addition, there was no significant difference in VAS and satisfaction scores after the examination.

**Table 5:** Incidence of postoperative adverse effects.

	Group N (*n* = 38)	Group E (*n* = 38)	p^*^
Nausea	0	0	>0.999
Vomiting	0	0	>0.999
Dizziness	18 (47.36)	24 (63.15)	0.166
Headache	0	0	>0.999
Drowsiness	8 (21.05)	13 (34.21)	0.200
Excessive dreams	3 (7.89)	5 (13.15)	0.709

Data are presented as *n* (%) and analyzed using chi-square test or Fisher’s exact test. ^*^Significance level (0.05).

## 4. Discussion

Esketamine is a right-handed split of ketamine, with an anesthetic effect twice as potent as ketamine (17, 23). Because of the dose-dependent side effects of ketamine, low-dose esketamine can reduce the incidence of anesthetic side reactions (24). At the equivalent dose of analgesia in healthy volunteers, esketamine has a lower incidence of psychotropic side effects than racemic ketamine, resulting in less impairment in concentration capacity and primary memory and fast recovery (9, 25). In this study, esketamine was applied with propofol for gastroscopy. The result showed that low dose of esketamine was generally effective and safe without obvious psychiatric adverse reactions.

In this study, we used 0.2 mg/kg esketamine and propofol for gastroscopy. Jie Wang found that both 0.5 mg/kg and 0.25 mg/kg esketamine reduced pain for cervical carcinoma patients after surgery (26). However, high dose of esketamine may result in undesirable effects. Francesca’s study showed that dosing of 0.3 mg/kg is possibly more effective than 0.15 mg/kg, but may be associated with adverse events like dizziness or lightheadedness (27). Another study revealed that incidences of adverse events were up to 75.0% in 0.5 mg/kg esketamine group for gastroscopy (17). In this trial, a subanesthetic dose of 0.2 mg/kg was selected in consideration of clinical effectiveness, safety and application convenience. Results showed that this dose provided satisfactory effect in gastroscopy and did not result in severe adverse reactions.

This study showed that 0.2 mg/kg esketamine significantly reduced total amount of propofol used for gastroscopy (21.7%). The result was similar to Feng’s study showing that EC50 (median effective concentration) of propofol when coadministration with 0.15, 0.25 or 0.5 mg/kg esketamine was decreased by 19.0, 32.2 and 61.5% compared with natural saline group during gastrointestinal endoscopy in adults (28). Zhan’s team also found that compared with group P (propofol+saline), propofol consumption per minute in groups PK2 (propofol+esketamine 0.1 mg/kg) and PK3 (propofol+esketamine 0.2 mg/kg) decreased by 13.92 and 18.76%. However, when concentration of esketamine decreased to 0.05 mg/kg, propofol consumption was not significantly different between propofol group and esketamine group.

The incidence of pain at the injection site after propofol application is up to 50–80% (29, 30). This is an undesirable feature of propofol, researchers are studying different medications and methods to relieve IV propofol pain, increase comfort during anesthesia, and improve patient satisfaction (31–33). Esketamine with analgesic effect significantly reduced injection pain by 61.5% in this study. The result was also proved by Tan’s research, which recommended a dose of 0.2 mg/kg IV esketamine before induction of anesthesia to reduce the pain of propofol injection. Another study showed that incidences of injection pain were not significantly different between 0.2 mg/kg esketamine group and propofol group. The possible reason may be low incidence of injection pain in this study. Only 3 patients in propofol group and 1 patient in esketamine group experienced injection pain (34).

Involuntary movements can disrupt attention and operation of the endoscopists. Frequent involuntary movements during examination may even cause severe injury to the gastrointestinal tract. Therefore, stable physical status without undesired movements was important in endoscopy. Maria Damps et al. found for children undergoing endoscopy of the upper gastrointestinal tract, the need to administer an additional dose of propofol (0.5 mg/kg) when child movement occurred was much less in ketamine group (10.9%) compared with remifentanil group (77.3%) (35). Zhan’s research also showed that compared with propofol group, propofol plus esketamine 0.2 mg/kg group showed less cough and body movement (34). The result was consistent with our study, where incidence of involuntary movement was significantly lower in group E than in group N, which provided more favorable condition for endoscopists, improved quality of gastroscopy and avoided potential harm to patients.

Previous studies showed lower incidence of hypotension when propofol was used with esketamine during gastrointestinal endoscopy in both younger and elder population (28, 36, 37). Similar result was also found in this study in which fewer patients in esketamine group experienced hypotension. More stable circulation may result from reduction of propofol and addition of esketamine, whose sympathetic-like effect partially reversed circulation inhibition of propofol. We also found that incidence of tachycardia was higher in the esketamine group, but no cases of palpitation or myocardial ischemia were reported. Similar result was also demonstrated in patients undergoing endoscopic retrograde cholangiopancreatography (ERCP), where esketamine did not increase cardiovascular adverse events (38).

In this study, there was no significant difference in total examination time or recovery time between two groups. Previous study showed that recovery time was significantly longer in esketamine 0.5 mg/kg group compared with esketamine 0.15, and 0.25 mg/kg groups (28). This trial revealed that subanesthetic dose of esketamine did not affect recovery time, which was suitable for examination or operation of short duration like gastroscopy. Another clinical study even found that combination medication of propofol with esketamine significantly shortened the recovery time in adults aging 65 years and above (37). This could be related to greater reduction of propofol amount in elderly patients.

The limitations of this trial were as follows. First, 0.2 mg/kg esketamine was used to compare with the control group. However, the optimal dose of esketamine for gastroscopy was unknown. We did not set a dosage ladder to verify this problem, which would be further explored in future study. Second, we used manual intravenous infusion for medication administration, which was not as accurate as target-controlled infusion. Future studies can apply target-controlled infusion and bispectral index-monitor for more precise outcome. Also, follow-up of some possible psychiatric symptoms was based on memory of the patient, which could cause recall bias. Last but not least, this study recruited patients aged 18–64 years, but 60 years or older may be different as younger patients. Further study can enlarge sample size and subdivide age groups to investigate detailed effect of esketamine.

## 5. Conclusion

Combination of propofol with 0.2 mg/kg esketamine reduced total amount of propofol, provided a more stable hemodynamic status and did not affect recovery time in gastroscopy.

## Data availability statement

The raw data supporting the conclusions of this article will be made available by the authors, without undue reservation.

## Ethics statement

The studies involving humans were approved by The Medical Ethics Committee of the First Affiliated Hospital of Shantou University Medical College. The studies were conducted in accordance with the local legislation and institutional requirements. The participants provided their written informed consent to participate in this study.

## Author contributions

XL and QX made substantial contributions to conception and design of the study. XL collected the data and performed the statistical analysis. QX wrote the first draft of the manuscript. SZ provided technical guidance. All authors contributed to the article and approved the submitted version.

## Conflict of interest

The authors declare that the research was conducted in the absence of any commercial or financial relationships that could be construed as a potential conflict of interest.

## Publisher’s note

All claims expressed in this article are solely those of the authors and do not necessarily represent those of their affiliated organizations, or those of the publisher, the editors and the reviewers. Any product that may be evaluated in this article, or claim that may be made by its manufacturer, is not guaranteed or endorsed by the publisher.
